# N-acetyl cysteine turns EPAC activators into potent killers of acute lymphoblastic leukemia cells

**DOI:** 10.1016/j.jbc.2023.105509

**Published:** 2023-12-01

**Authors:** Nina Richartz, Wojciech Pietka, Ajay Yadav, Monica Bostad, Sampada Bhagwat, Soheil Naderi, Elin Hallan Naderi, Trond Stokke, Ellen Ruud, Heidi Kiil Blomhoff

**Affiliations:** 1 Department of Molecular Medicine, Institute of Basic Medical Sciences, University of Oslo, Oslo, Norway; 2 Department of Core Facilities, Norwegian Radium Hospital, Oslo University Hospital, Oslo, Norway; 3 Division of Laboratory Medicine, Department of Pharmacology, Oslo University Hospital, Oslo, Norway; 4 Section of Head and Neck Oncology, Department of Oncology, Oslo University Hospital, Oslo, Norway; 5 Division of Paediatric and Adolescent Medicine, Oslo University Hospital, Oslo, Norway; 6 Faculty of Medicine, Institute of Clinical Medicine, University of Oslo, Oslo, Norway

**Keywords:** cyclic AMP (cAMP), cell death, apoptosis, irradiation, leukemia, calcium, reactive oxygen species (ROS), autophagy, animal models

## Abstract

Today, the majority of patients with pediatric B cell precursor acute lymphoblastic leukemia (BCP-ALL, hereafter ALL) survive their disease, but many of the survivors suffer from life-limiting late effects of the treatment. ALL develops in the bone marrow, where the cells are exposed to cAMP-generating prostaglandin E_2_. We have previously identified the cAMP signaling pathway as a putative target for improved efficacy of ALL treatment, based on the ability of cAMP signaling to reduce apoptosis induced by DNA damaging agents. In the present study, we have identified the antioxidant N-acetyl cysteine (NAC) as a powerful modifier of critical events downstream of the cell-permeable cAMP analog 8-(4-chlorophenylthio) adenosine-3′, 5′- cyclic monophosphate (8-CPT). Accordingly, we found NAC to turn 8-CPT into a potent killer of ALL cells *in vitro* both in the presence and absence of DNA damaging treatment. Furthermore, we revealed that NAC in combination with 8-CPT is able to delay the progression of ALL in a xenograft model in NOD-*scid* IL2Rγ^null^ mice. NAC was shown to rely on the ability of 8-CPT to activate the guanine-nucleotide exchange factor EPAC, and we demonstrated that the ALL cells are killed by apoptosis involving sustained elevated levels of calcium imposed by the combination of the two drugs. Taken together, we propose that 8-CPT in the presence of NAC might be utilized as a novel strategy for treating pediatric ALL patients, and that this powerful combination might be exploited to enhance the therapeutic index of current ALL targeting therapies.

B cell precursor acute lymphoblastic leukemia (BCP-ALL, hereafter ALL) is the most common type of pediatric cancer (1). Gradual improvements in multimodal therapy regimens have increased the survival rate of patients with ALL to almost 90%. However, despite these significant advancements, ALL is still the most frequent cause of cancer death in children (2, 3). Moreover, ALL survivors encounter long-term morbidity due to adverse effects of the treatment that commonly includes DNA-damaging drugs. Accordingly, new therapeutic strategies are still highly required.

ALL cells develop in the bone marrow in close contact with bone marrow-derived stromal cells (4). These stromal cells are known to release signaling molecules that may influence cancer growth and lead to reduced treatment efficacy (5, 6). Prostaglandin E_2_ (PGE_2_) is one of the factors released by bone marrow-derived stromal cells (7, 8). PGE_2_ is known to bind to prostanoid receptor 2 (EP2) expressed on leukemic cells, resulting in activation of adenylyl cyclase and increased levels of endogenous adenosine-3′,5′-cyclic monophosphate (cAMP) (9, 10). We have previously highlighted the role of the cAMP/protein kinase A (PKA) pathway in protecting ALL cells from DNA damage-induced responses such as p53-mediated apoptosis (7, 11, 12, 13). In addition, we showed that inhibiting PGE_2_ by indomethacin slowed down the leukemia progression in a human ALL xenograft model (14).

Although the classical effector of the second messenger cAMP is PKA, cAMP is also known to activate the exchange protein directly activated by cAMP (EPAC) independent of PKA (15). EPAC exists in two isoforms EPAC1 and EPAC2. Both isoforms are guanine-nucleotide exchange factors for the Ras-like GTPases, Rap1 and Rap2 (16, 17). Like PKA, EPACs seem to have pleiotropic effects depending on tissue type and context. There are conflicting reports regarding the role of EPACs in cancer development, linked to both the promotion and inhibition of cancer progression (18). For instance, EPAC1 has been implicated in promoting cell migration and metastasis of melanoma (19), whereas genetic or pharmacological inhibition of EPAC1 has been reported to reduce metastasis of pancreatic cancer (20, 21). At the mechanistic level, EPAC appears to act in an anti-apoptotic manner in T-ALL and in B chronic lymphocytic leukemia cells (22, 23), in contrast to having a pro-apoptotic role in the murine immature B-cell lymphoma cell line WEH1 (24). There have been few attempts to explain the differential effects of EPACs on apoptosis at the molecular level. EPACs are known to activate the Rap1/H-Ras/ERK/Akt pathway (23, 24), which may promote cell survival. In addition, EPACs can also activate phospholipase C (PLC), resulting in inositol triphosphate (IP_3_) release and activation of calcium signaling (19, 25). Importantly, extensive calcium signaling over time may result in apoptotic cell death (26).

We have previously shown that cAMP signaling induced by the adenylyl cyclase activator forskolin protects ALL cells from DNA damage-induced apoptosis by enhancing autophagy (27). In a recent study, we revealed that the forskolin-mediated autophagy involves activation of poly (ADP-ribose) polymerase 1 (PARP1) (28). Moreover, we showed that the reactive oxygen species (ROS) scavenger N-acetyl cysteine (NAC) reduced PARP1 activation, prevented the forskolin-induced autophagy, and reduced the protective effect of cAMP signaling on DNA damage-induced apoptosis. In the present paper, we aimed to advance our understanding of the interplay between NAC and cAMP signaling in regulating cell death. As a first attempt, we sought an alternative way of stimulating the cAMP signaling pathway, by including the cAMP analogue 8-(4-chlorophenylthio) cAMP (8-CPT-cAMP, hereafter 8-CPT). As expected, 8-CPT reduced the DNA damage-induced killing of ALL cells in the same manner as forskolin, and we therefore anticipated that NAC would diminish the protective effects of 8-CPT in the same mode as it reduced the forskolin-mediated protection. It turned out that NAC had a significantly stronger effect on 8-CPT than on forskolin-mediated protection, resulting in enhanced DNA damage-induced cell death. Moreover, we showed that whereas NAC had no effect on forskolin alone, NAC converted 8-CPT into a potent killer of ALL cells also in the absence of DNA-damaging agents. 8-CPT is known to activate both PKA and EPACs (15), and we here reveal how NAC selectively utilizes EPAC activators to kill ALL cells both *in vitro, ex vivo,* and *in vivo.*

## Results

### NAC turns 8-CPT into a potent killer of REH cells

**Figure 1:**
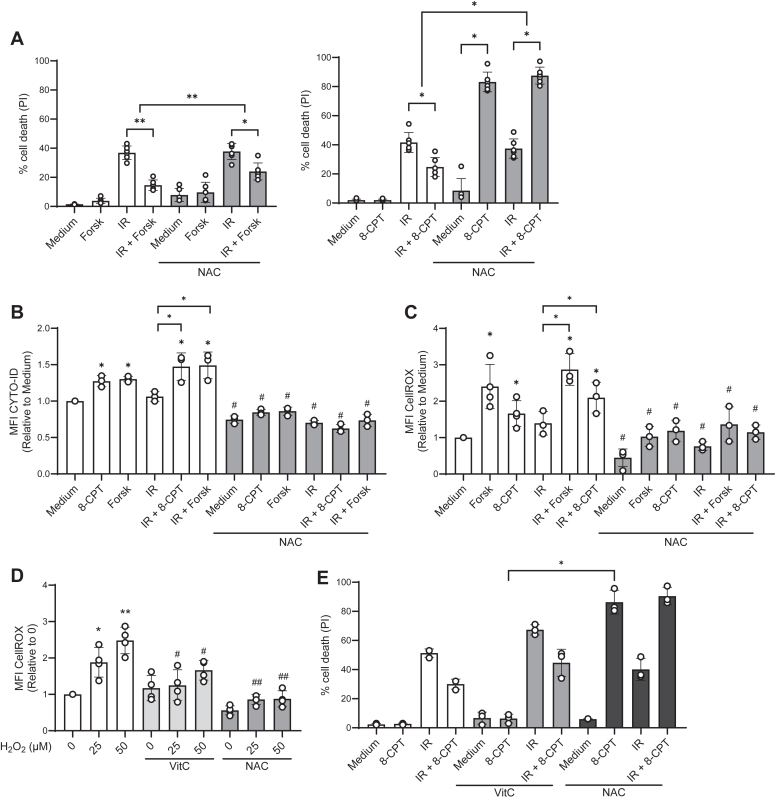
**NAC turns 8-CPT from a protector into an enhancer of IR-induced cell death.***A*, REH cells (4 × 10^5^ cells/ml) were incubated in the presence or absence of 30 mM NAC for 30 min, followed by incubation with forskolin (Forsk, 60 μM) (*left panel*) or 100 μM 8-CPT (*right panel*) for 45 min prior to IR (10 Gy). PI-staining was performed 24 h after IR, analyzed by flow cytometry and shown as percentage of PI-positive cells (n = 6, ∗*p* < 0.05, ∗∗*p* < 0.01, one-way ANOVA). *B*, REH cells (4 × 10^5^ cells/ml) were treated in the presence or absence of NAC, forskolin, 8-CPT and IR as described in Figure 1*A*. Autophagy was determined 24 h after IR by CYTO-ID staining, and the results are presented relative to the level in untreated cells (n = 3, ∗*p* < 0.05, #*p* < 0.05 relative to sample treated without NAC, one-way ANOVA). *C*, REH cells (4 × 10^5^ cells/ml) were treated in the presence or absence of NAC, forskolin, 8-CPT and IR as described in Figure 1*A*. Cells were stained with CellROX green 2 h after IR (n = 3, ∗*p* < 0.05, #*p* < 0.05 relative to the sample treated without NAC, one-way ANOVA). *D*, Reh cells (4 × 10^5^ cells/ml) were incubated in the presence or absence of Vitamin C (Vit C, 100 μM) or NAC (10 mM) for 1 h, followed by treatment with H_2_O_2_ (25 or 50 μM) for 30 min. ROS levels were analyzed by flow cytometry using the CellROX Green reagent from ThermoFisher (n = 4, ∗*p* < 0.05, ∗∗*p* < 0.01, one-way ANOVA). *E*, Reh cells (4 × 10^5^ cells/ml) were incubated with VitC (100 μM) for 1 h, followed by treatment with 8-CPT (100 μM) for 45 min, then irradiation (IR, 10 Gy). Cell death was analyzed by flow cytometry with PI-staining after 24 h (n = 3, ∗*p* < 0.01, one-way ANOVA).

We recently established that cAMP signaling *via* the adenylyl cyclase activator forskolin protects ALL cells from X-ray irradiation (IR)-induced apoptosis *via* ROS-mediated autophagy (28). Accordingly, we aimed to reveal the mechanisms linking elevated cAMP levels to the downstream events. To this end, we treated the ALL-derived REH cells with the cAMP analog 8-CPT. 8-CPT was originally regarded as a specific PKA activator (29), but has later also been identified as a potent activator of EPACs (30). For comparisons, we included forskolin in our experiments, the compound that we routinely have used as a stimulator of cAMP signaling in ALL cells (12, 27). Both 8-CPT and forskolin were added to cell cultures prior to irradiation. After 24 h, cell death, autophagy and ROS levels were assessed by staining with propidium iodide (PI), CYTO-ID, or with the CellROX green reagent, respectively. In line with our previous results (11, 27), both 8-CPT and forskolin inhibited the IR-induced cell death (Fig. 1*A*) and enhanced the IR-induced autophagy (Fig. 1*B*). However, compared to forskolin, 8-CPT was a significantly weaker ROS inducer (Fig. 1*C*).

To address the difference between forskolin- and 8-CPT-induced ROS levels, we treated the REH cells with the ROS scavenger N-acetyl-L-cysteine (NAC), 30 min prior to exposure to forskolin or 8-CPT. NAC had no effect on the forskolin-mediated cell death, and partially relieved the forskolin-mediated protection against IR-induced cell death (Fig. 1*A*, left panel). In contrast, NAC in the presence of 8-CPT killed 80% of the cells, and it killed nearly 90% of the REH cells exposed to 8-CPT in combination with IR (Fig. 1*A*, right panel). Notably, however, there were no differential effects of NAC on forskolin-*versus* 8-CPT-induced autophagy (Fig. 1*B*) or on ROS levels (Fig. 1*C*). Thus, NAC reduced both the autophagy and ROS levels induced by forskolin as well as by 8-CPT, both in the presence and the absence of IR.

Taken together, the present results show that NAC selectively turns 8-CPT into a potent killer of ALL cells, both in the absence and presence of IR. Furthermore, our results suggest that neither changes in autophagy nor in ROS levels can explain this pronounced induction of cell death (Fig. 1, *B* and *C*). To further verify that NAC kills the 8-CPT-treated cells independent of its antioxidant ability, we examined the effect of the ROS scavenger ascorbic acid (vitamin C). As shown in Figure 1*D*, both NAC and vitamin C reduced the ROS levels induced by hydrogen peroxide. Still, only NAC and not vitamin C enhanced the 8-CPT-induced cell death in the presence or absence of IR (Fig. 1*E*).

### NAC in combination with 8-CPT enhances cell death induced by IR and doxorubicin

**Figure 2:**
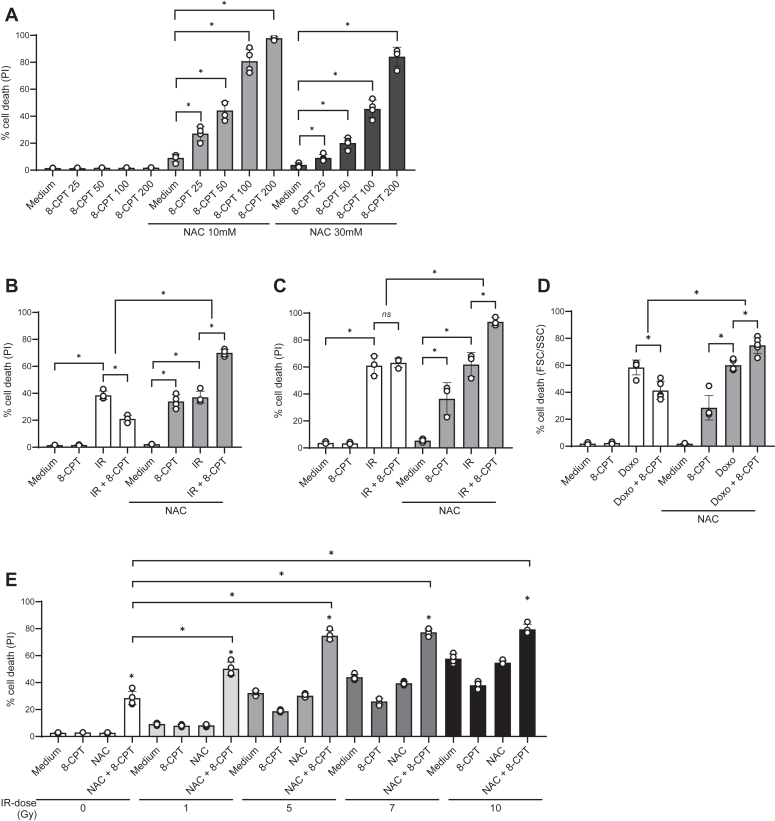
**Combination of NAC and 8-CPT potentiates IR- and doxorubicin-induced cell death.***A*, REH cells (4 × 10^5^ cells/ml) were incubated in the presence or absence of 10 mM and 30 mM NAC for 30 min, followed by incubation with 25 to 200 μM 8-CPT. PI-staining was performed 24 h after addition of 8-CPT, analyzed by flow cytometry and shown as percentage of PI-positive cells (n = 4, ∗*p* < 0.01, one-way ANOVA). *B*, REH cells (4 × 10^5^ cells/ml). *C*, NALM-6 cells (4 × 10^5^ cells/ml) were incubated in the presence or absence of 10 mM NAC for 30 min, followed by incubation with 100 μM 8-CPT for 45 min prior to IR (10 Gy). PI-staining was performed 24 h after IR, analyzed by flow cytometry and shown as percentage of PI-positive cells (n = 3–4, ∗*p* < 0.05, one-way ANOVA. *D*, REH cells (4 × 10^5^ cells/ml) were incubated in the presence or absence of 10 mM NAC for 30 min, followed by incubation with 100 μM 8-CPT for 45 min prior to treatment with 100 nM doxorubicin. Flow cytometry analysis was performed 24 h after the addition of doxorubicin, and the results are shown as a percentage of dead cells based on low forward scatter (FSC) and high side scatter (SSC) (n = 5, ∗*p* < 0.01, one-way ANOVA). *E*, REH cells (4 × 10^5^ cells/ml) were incubated in the presence or absence of 10 mM NAC for 30 min, followed by incubation with or without 100 μM 8-CPT and irradiated with the indicated doses of IR (1–10 Gy). PI staining was done 24 h after IR and analyzed by flow cytometry for PI-positive cells (n = 3, ∗*p* < 0.05, one-way ANOVA).

The ability of NAC together with 8-CPT to kill ALL cells rapidly and efficiently might have an important clinical impact by itself. However, we also wished to unravel the possibility of combining these compounds with conventional DNA-damaging therapeutics. The rationale behind this strategy would be to enhance the efficacy of the conventional ALL treatment, and thereby enable the use of lower doses of the DNA-damaging drugs that usually induce severe long-term morbidity in the patients (3). In the experiments presented in Figure 1, we noted that 30 mM of NAC in combination with 100 μM of 8-CPT was such a potent killer of the REH cells that hardly any potentiating effects on IR-mediated cell death could be detected (Fig. 1*A*). We therefore performed dose–response studies to choose the optimal concentrations of both NAC and 8-CPT to allow for potentiating effects on DNA damage-induced cell death (Fig. 2*A*). Accordingly, we chose 10 mM NAC together with 100 μM 8-CPT as the optimal combination for enhancing the killing of REH cells in the presence of 10 Gy of IR. As shown in Figure 2*B*, this treatment increased the IR-induced cell death from approximately 40% to nearly 70%. Next, we validated our findings in another acute lymphoblastic leukemia cell line, NALM-6, confirming that NAC and 8-CPT enhanced the IR-induced killing of ALL cells (Fig. 2*C*).

In our study, we have used irradiation as a model for assessing the role and regulation of DNA-damaging therapy of patients with ALL. Radiation therapy of ALL is in the clinic mainly used for localized treatment of recurring disease with central nervous system or testicular involvement. The clinical relevance of our results was therefore strengthened by using the cytotoxic drug doxorubicin as the DNA-damaging agent. Accordingly, we showed that NAC turned 8-CPT from an inhibitor of doxorubicin-induced killing of REH cells into a potent enhancer of drug-induced cell death (Fig 2*D*). Of clinical importance was also the finding that NAC together with 8-CPT was able to enhance the killing of suboptimal concentrations of the DNA damaging agent. Thus as shown in Figure 2*E*, NAC and 8-CPT notably enhanced the killing of ALL cells at irradiation doses as low as 1 Gy. Taken together, these results suggest that the combination of NAC and 8-CPT may potentiate the cytotoxicity of anti-leukemic therapy.

### NAC in combination with 8-CPT kills ALL cells *via* apoptosis

**Figure 3:**
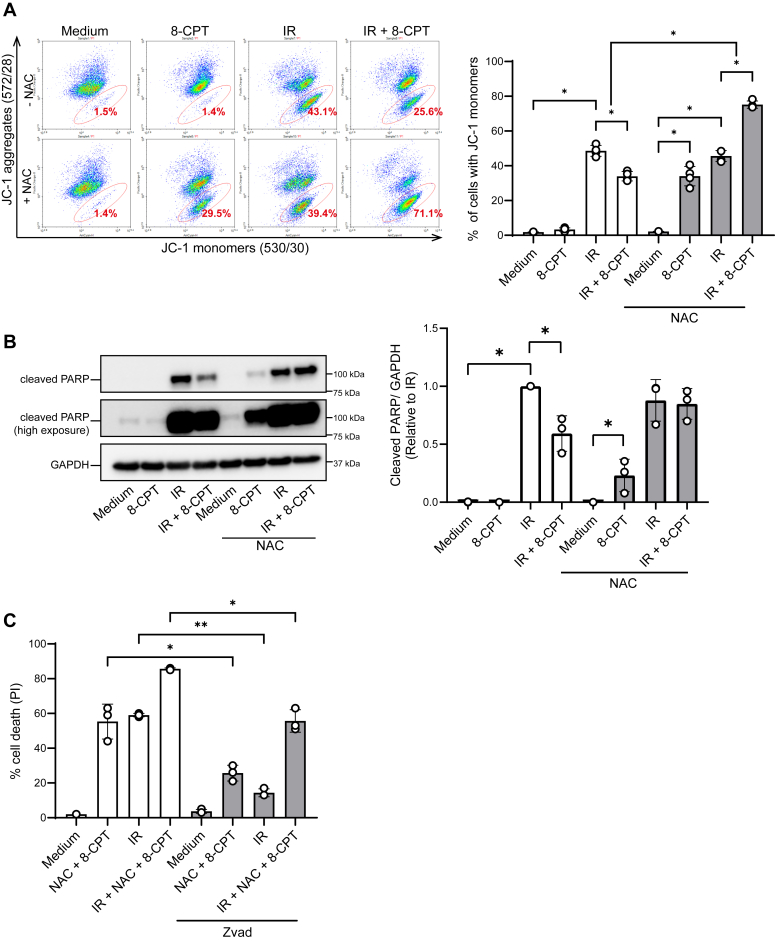
**8-CPT in the presence of NAC kills ALL cells *via* apoptosis.***A*, REH cells (4 × 10^5^ cells/ml) were incubated in the presence or absence of 10 mM NAC for 30 min, followed by incubation with 100 μM 8-CPT for 45 min prior to IR (10 Gy). Changes in mitochondrial membrane potential were assessed by JC-1-staining 24 h after IR. The *left panel* shows the gating strategy, with the apoptotic cell population being highlighted. The *right panel* represents quantification of JC-1 experiments (n = 4, ∗*p* < 0.01, one-way ANOVA). *B*, REH cells (1 × 10^6^ cells/ml) were incubated in the presence or absence of 10 mM NAC for 30 min, followed by incubation with 100 μM 8-CPT for 45 min prior to IR (10 Gy). Cells were harvested after 12 h. Total lysates (3 × 10^6^ cells) were subjected to immunoblot analyses with antibodies against cleaved-PARP and GAPDH. *Left panel* shows a representative Western blot. *Right panel* shows a quantified overview of three independent experiments, data are shown as a ratio of cleaved-PARP to GAPDH, normalized to the IR-only treated samples (n = 3, ∗*p* < 0.05, one-way ANOVA). *C*, REH cells (4 × 10^5^ cells/ml) were incubated with 100 μM Z-VAD for 30 min, followed by treatment with 100 μM 8-CPT for 45 min, then irradiation at 10 Gy. PI-staining was performed 24 h after IR, analyzed by flow cytometry and shown as percentage of PI-positive cells (n = 3, ∗*p* < 0.05, ∗∗*p* < 0.01, one-way ANOVA).

Having established that 8-CPT in the presence of NAC efficiently kills ALL cells, our next aim was to elucidate the mechanism involved. We have previously established that IR kills ALL cells *via* apoptosis, and that forskolin-induced cAMP levels inhibit apoptosis *via* both p53-dependent- and p53-indpendent mechanisms (7, 11, 12, 27, 28). To address the mode of cell death induced by 8-CPT and NAC, we took advantage of features that are characteristic of apoptosis, namely, loss of mitochondrial membrane potential and cleavage of the caspase substrate poly (ADP-ribose) polymerase (PARP) (31). To assess the changes in mitochondrial membrane potential, we stained the cells with the cyanine dye JC-1. JC-1 accumulates in the mitochondria and forms red fluorescent aggregates in viable cells, and green monomers in apoptotic cells (32). The conclusion was that 8-CPT in the absence of NAC reduced the IR-induced apoptosis from 42% to 26%, whereas 8-CPT in the presence of NAC enhanced the IR-induced apoptosis from 42% to 72% (Fig. 3*A*). The mode of cell death was further validated by assessing the activation of caspases after 12 h. To this end, we determined the cleavage of PARP by Western blot analyses. As shown in Figure 3*B*, no cleavage of PARP was noted by 8-CPT alone, and the level of IR-mediated cleavage of PARP was as expected reduced by 8-CPT. In contrast, NAC significantly enhanced the levels of 8-CPT-induced cleavage of PARP and reversed the 8-CPT-mediated inhibition of IR-induced PAPRP cleavage. Finally, we showed that the caspase inhibitor Z-VAD markedly reduced the cell death induced by NAC and 8-CPT in the presence and absence of IR (Fig. 3*C*). Together, these data strongly suggest that 8-CPT in the presence of NAC kills leukemic cells *via* apoptosis.

### 8-CPT in the presence of NAC kills ALL cells *via* EPAC

**Figure 4:**
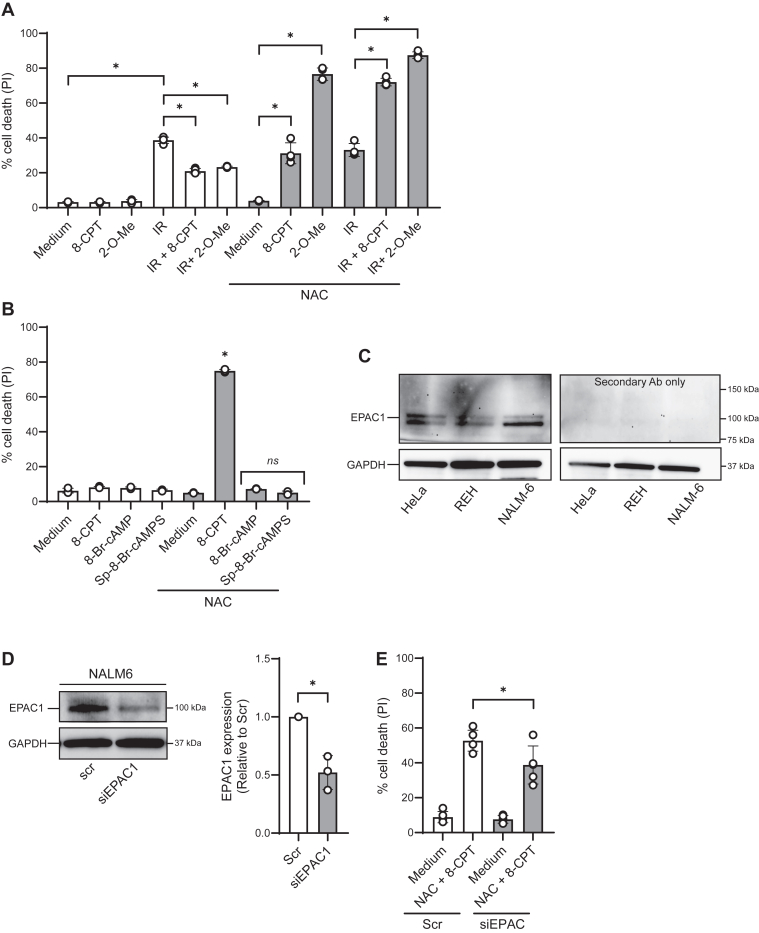
**8-CPT in the presence of NAC kills ALL cells *via* EPAC.***A*, REH cells (4 × 10^5^ cells/ml) were incubated in the presence or absence of 10 mM NAC for 30 min, followed by incubation with 100 μM 8-CPT or 8-pCPT-2′-O-Me-cAMP for 45 min prior to IR (10 Gy). PI staining was performed 24 h after IR, analyzed by flow cytometry and shown as percentage of PI-positive cells (n = 4, ∗*p* < 0.01, one-way ANOVA). *B*, REH cells (4 × 10^5^ cells/ml) were incubated with 10 mM NAC for 30 min, followed by incubation with 100 μM 8-CPT, 8-Br-cAMP, or Sp-8-Br-cAMPS. PI staining was performed after 24 h (n = 3, ∗*p* < 0.01, one-way ANOVA). *C*, REH and NALM-6 cell lysates (3 × 10^6^ cells) were subjected to immunoblot analyses with antibodies against EPAC1 (*upper left panel*) and GAPDH (*lower panels*). To verify specificity of the EPAC1 antibody, the membrane was incubated with secondary antibody only (*upper right panel*). *D*, NALM-6 cells (3 × 10^6^ cells) were transfected with siRNA against EPAC1 (or with scrambled siRNA (Scr) as control) as described in Experimental procedures. Cells were harvested after 72 h for knockdown validation. *Left panel* depicts the Western blot image of EPAC knock-down in NALM-6 cells, while the *right panel* shows quantitation of the knock-down in three independent experiments (∗*p* < 0.05, paired *t* test). *E*, NALM-6 cells were exposed to siRNA- or scrambled-treatment prior to NAC and 8-CPT treatment. PI staining was performed 24 h after 8-CPT treatment (n = 5, ∗*p* < 0.05, one-way ANOVA).

The experiments presented in Figure 1, suggested that the NAC/8-CPT-induced killing of ALL cells does not involve ROS-mediated autophagy. To explore alternative mechanisms, we first examined the possibility that NAC interacts with EPAC-specific signaling. Since 8-CPT is known to activate both PKA and EPACs (15), we assessed the effect of the EPAC-specific cAMP analog 2′-O-Methyl-cAMP (2′-O-Me-cAMP, hereafter 2′-O-Me) (33) on cell death in REH cells. When measured after 24 h, 2′-O-Me in the presence of NAC was an even stronger inducer of cell death than the equimolar concentration of 8-CPT. NAC enhanced the cell death induced by 2′-O-Me from 5% to approximately 75%, whereas the combination of NAC and 8-CPT resulted in 35% of dying cells (Fig. 4*A*). Moreover, IR-mediated cell death was enhanced by the combination of NAC and 2′-O-Me-cAMP from 32% to nearly 90% (Fig. 4*A*). By demonstrating that NAC did not affect the cell death induced by the PKA-specific cAMP analogues 8-Br-cAMP and Sp-8-Br-cAMP (Fig. 4*B*), we verified that the ability of NAC to enhance the 8-CPT-mediated cell death was mediated *via* EPAC and not *via* PKA.

We confirmed the expression of EPAC1 in the ALL-derived cell lines REH and NALM-6 by Western blot analysis (Fig. 4*C*). As a positive control, we included HeLa cells, which according to the Human Protein Atlas are known to express EPAC1. To establish the role of EPAC1 in the NAC/8-CPT-mediated killing of the ALL cells, we reduced the levels of EPAC1 by siRNA-mediated knockdown. Since the transfection efficiency was higher in NALM-6 cells compared to REH cells, the knockdown experiments were performed on NALM-6. As shown in Figure 4*C*, we obtained approximately 50% reduction in the levels of EPAC1 by siRNA, and this resulted in a decrease in NAC/8-CPT-induced killing of NALM-6 cells by approximately 30% (Fig. 4*D*). Taken together, our results indicate that EPAC1 is involved in the 8-CPT-mediated killing of ALL cells in the presence of NAC.

### Calcium is an important mediator of apoptosis induced by 8-CPT in the presence of NAC

To address the mechanism whereby NAC could turn EPAC-agonists into potent killers of ALL cells, we explored the signaling events downstream of EPACs and NAC. It has been reported that both NAC and EPACs can enhance the calcium flux in cells like melanoma (19) and neutrophils (34). Since disruption of calcium homeostasis is associated with induction of apoptosis (35), we hypothesized that NAC-mediated calcium flux could intensify EPAC-mediated release of calcium from internal stores, culminating in calcium overload and resulting in apoptosis.

**Figure 5:**
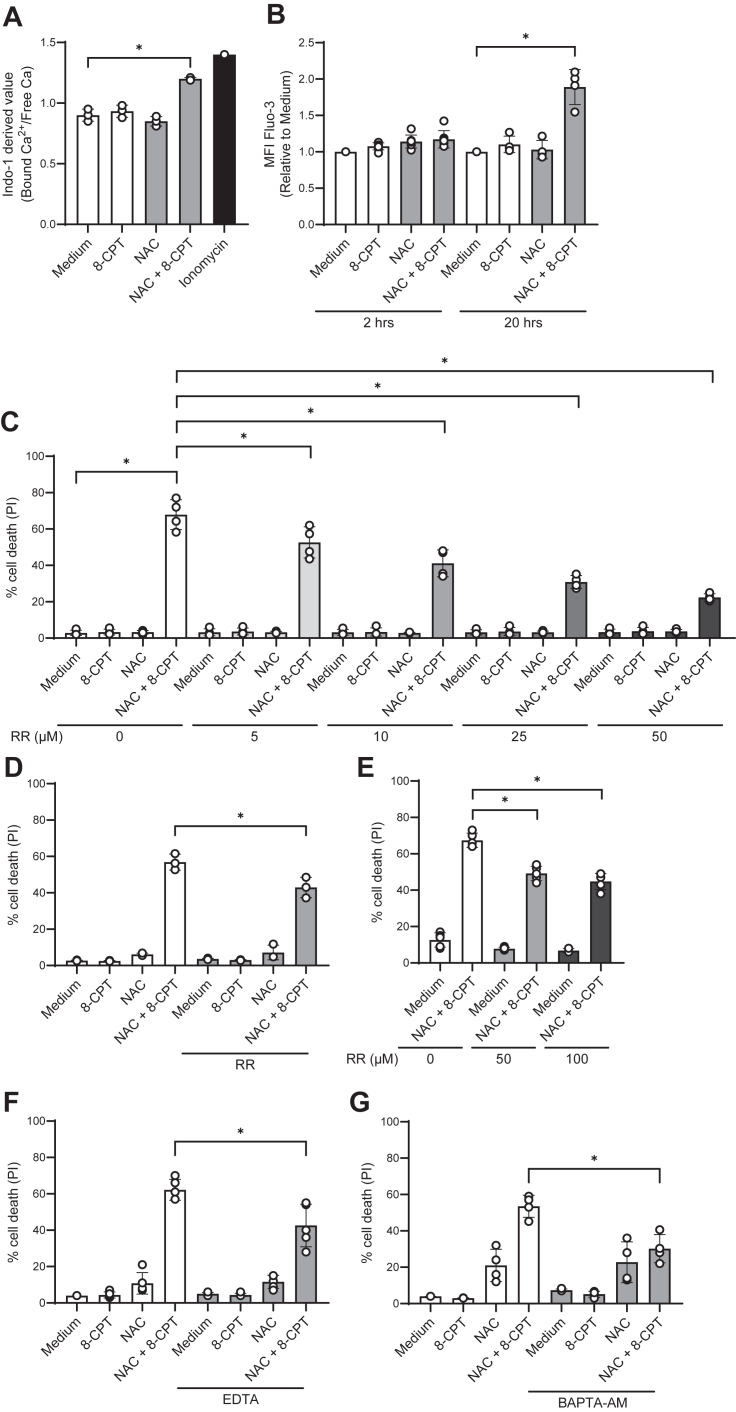
**Calcium is an important mediator of NAC and 8-CPT-induced apoptosis.***A*, REH cells (4 × 10^5^ cells/ml) were pre-treated with or without 10 mM NAC for 30 min, then incubated with or without 100 μM 8-CPT for 20 h, followed by Indo-1 staining and flow cytometry analysis. The Indo-1 derived value is the ratio of bound calcium (405 nm emission) to free calcium (485 nm emission). Data are shown as mean value ± S.D., n = 3. ∗*p* < 0.05 (paired *t* test). *B*, REH cells (4 × 10^5^ cells/ml) were incubated with or without NAC and 8-CPT as in Figure 5*A*. Fluo-3 staining was performed at 2 h and 20 h after 8-CPT addition, followed by flow cytometry analysis. Numerical values are presented as mean fluorescence intensity (MFI), normalized to untreated sample at each time point. Data on the graph are shown as mean ± S.D., n = 4 to 7. ∗*p* < 0.01 (paired *t* test). *C*, REH cells, (*D*) NALM-6 cells, and (*E*) 697 cells, at 4 × 10^5^ cells/ml were pre-treated for 30 min with the inhibitor ruthenium red (RR) at concentrations 5 to 50 μM (*C*), 50 μM (*D*) and 50 to 100 μM (*E*) prior to NAC and 8-CPT treatment as described in Figure 5*A*. *F*, REH cells and (*G*) NALM-6 cells (4 × 10^5^ cells/ml) were pre-treated with calcium chelators, 500 μM EDTA (*F*) and 1 μM BAPTA AM (*G*), 30 min prior to NAC and 8-CPT treatment as described in Figure 5*A*. *C*–*G*, PI staining was performed 24 h after 8-CPT treatment, analyzed by flow cytometry and shown as percentage of PI-positive cells (n = 4–5. ∗*p* < 0.05, one-way ANOVA).

To test our hypothesis, we measured the effects of 8-CPT and NAC on intracellular calcium levels in REH cells by using two different calcium flux indicators, Indo-1 (Fig. 5*A*) and Fluo-3 (Fig. 5*B*). We did not observe any effects of 8-CPT and NAC on calcium levels at early time points (<5 min) (data not shown). We therefore turned to study calcium fluxes at later time points, keeping in mind that moderate, but sustained elevated levels of calcium may result in apoptosis (35). When using Fluo-3, we noted small, but reproducible effects of NAC and 8-CPT after 2 h (Fig. 5*B*). Furthermore, we revealed that the combination of NAC and 8-CPT markedly enhanced calcium levels 20 h after addition of both Indo-1 and Fluo-3 (Fig. 5, *A* and *B*).

To further address the role of calcium in the NAC/8-CPT-mediated cell death, ALL cells were pre-treated with various forms of calcium channel inhibitors or calcium chelating agents before measuring the cell death after 24 h. As shown in Figure 5*C*, the calcium channel inhibitor Ruthenium red (RR) reduced the NAC/8-CPT-induced cell death in a dose-dependent manner, without being toxic by itself. Thus at 50 μM of RR, the percentage of dead REH cells was reduced from 75% to approximately 20%. The effect of RR was less pronounced in the two ALL-derived cell lines NALM-6 (Fig. 5*D*) and 697 (Fig. 5*E*), but RR still reduced the cell death in both cell lines by almost 30%. The non-cell permeable calcium-chelating agent EDTA reduced the NAC/8-CPT-induced killing of REH cells by 35% (Fig. 5*F*), whereas pre-treatment of NALM-6 with the calcium chelator BAPTA-AM reduced the NAC/8-CPT-induced cell death by nearly 55% (Fig. 5*G*). Taken together, our results suggest that apoptosis induced by 8-CPT in the presence of NAC is mediated *via* calcium.

### cAMP kinetics in relation to NAC-induced cell death

**Figure 6:**
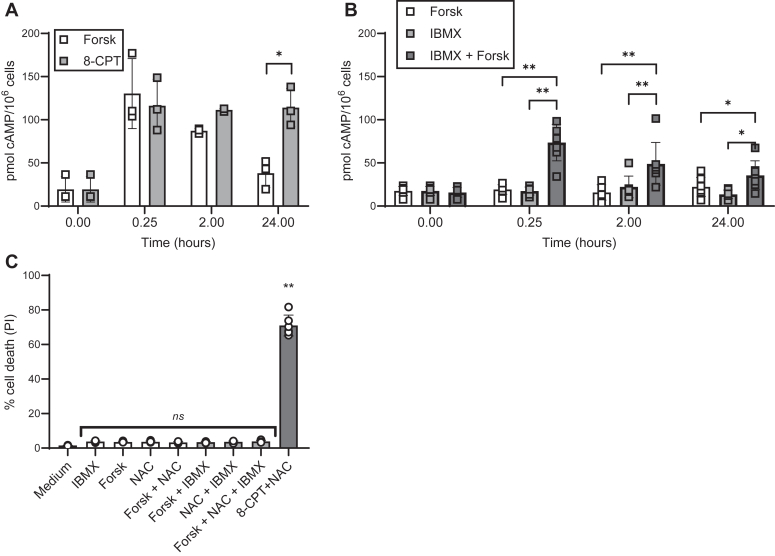
**No correlation between cAMP kinetics and the ability to kill ALL cells.***A*, REH cells (1 × 10^6^ cells/ml) were incubated with forskolin (Forsk, 60 μM) or 8-CPT (100 μM) (n = 5, ∗*p* < 0.01, one-way ANOVA). *B*, REH cells (1 × 10^6^ cells/ml) were incubated with 100 μM IBMX and/or 10 μM forskolin(n = 7, ∗∗*p* < 0.01, ∗*p* < 0.05, one-way ANOVA) (*A* and *B*) At the indicated time points, 1 × 10^6^ cells were collected, and cAMP levels measured as described in the Experimental procedures section. *C*, REH cells (4 × 10^5^ cells/ml) were pre-incubated with 10 mM NAC, followed by incubation with 100 μM IBMX and/or 10 μM Forskolin. Cells treated with 100 μM 8-CPT and 10 mM NAC were used as positive control. PI-staining was performed after 24 h, followed by flow cytometry analysis of PI-positive cells (n = 6. ∗∗*p* < 0.01, one-way ANOVA).

Knowing that both 8-CPT and forskolin act by enhancing the intracellular levels of cAMP, we reasoned that the differential effects of NAC on 8-CPT- and forskolin-mediated apoptosis could be due to differences in the kinetics of cAMP induction by the two compounds. Accordingly, we measured the intracellular levels of cAMP induced by either forskolin or 8-CPT. As shown in Figure 6*A*, both forskolin and 8-CPT induced a rapid (within 15 min) induction of cAMP. However, whereas the forskolin-induced cAMP levels were transient and were reduced to background levels within the next 24 h, the 8-CPT-induced cAMP levels remained elevated for the whole 24 h treatment period. Taken together, these results could imply that the sustained elevated cAMP levels induced by 8-CPT might explain the massive cell death in the presence of NAC.

To test our hypothesis, we prolonged the cAMP levels induced by forskolin by adding the phosphodiesterase inhibitor 1-Methyl-3-isobutylxanthine (IBMX). As shown in Figure 6*B*, the cAMP levels induced by low levels (10 μM) of forskolin in combination with IBMX remained high throughout the 24 h treatment period. However, despite these prolonged elevated levels of cAMP, NAC was not able to enhance the cell death induced by IBMX and forskolin (Fig. 6*C*). Taken together, these results imply that the differential effects of NAC on 8-CPT *versus* forskolin-induced cell death cannot be explained by the differences in the duration of increase in cAMP levels induced by the two compounds.

### NAC together with 8-CPT kills primary patient-derived ALL cells *ex vivo*

**Figure 7:**
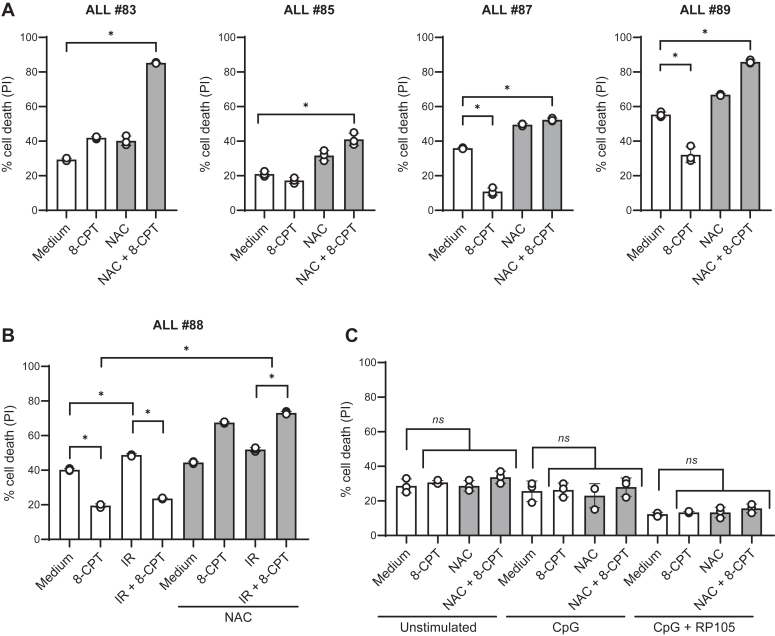
**NAC enhances 8-CPT-induced cell death in primary ALL cells.***A* and *B*, leukemic cells freshly isolated from bone marrow aspirates of patients with ALL were treated with 10 mM NAC for 30 min followed by 8-CPT at doses of 100 or 200 μM. B, ALL#88 cells were in addition treated with IR (5 Gy) after 45 min incubation with 8-CPT. PI staining was performed 24 h after 8-CPT treatment (*A*) or IR treatment (*B*) (n = 3 technical replicates, ∗*p* < 0.05, one-way ANOVA). *C*, B cells were isolated from the buffycoats of healthy human blood donors and stimulated with or without 1 μg/ml CpG-ODN and anti-RP105 for 24 h, followed by treatment with 100 μM 8-CPT and 10 mM NAC as described in Figure 7*A*. PI staining was performed 24 h after treatment (n = 4).

The clinical relevance of our findings was strengthened by including primary leukemic cells collected from patients diagnosed with ALL (patient characteristics described in Table 1). As presented in Figure 7, the viability of the primary ALL cells cultured for 24 h varied from 45% to 80%, presumably reflecting both inborn differences in cell vulnerability, and differences that might occur in handling the bone marrow samples during the harvesting procedure.

**Table 1:** Patient characteristics

**Patient sample**	ALL#83	ALL#85	ALL#87	ALL#88	ALL#89
Age, years	8	4	5	8	2
Sex	M	F	F	F	F
Bone marrow infiltration at diagnosis (% CD19^+^/CD10^+^)	92%	86%	92%	75%	67%
Cytogenetics	t(1;19)(q23;p13)	Normal karyotype	Hyperdiploid	t(9;22)(q34;q11)	Hyperdiploid

In cells from patients #85, #87, #88, and #89 with ALL, 200 μM of 8-CPT alone enhanced the viability of the cells, whereas 8-CPT in the presence of NAC was turned into a potent killer of the cells (Fig. 7, *A* and *B*). In cells from ALL #83 the effect of 8-CPT and NAC was so potent that we reduced the concentration of 8-CPT to 100 μM to avoid too much cell death. We also examined the effect of 8-CPT and NAC on IR-mediated cell death in leukemic cells from patient #88. As shown in Figure 7*B*, the IR-induced cell death was enhanced from approximately 50% to 70% in the presence of NAC and 8-CPT. Taken together, these results suggest that 8-CPT in the presence of NAC efficiently kills primary ALL cells, and that this combination of drugs also has the potential to enhance the effects of DNA-damaging agents.

### NAC in combination with 8-CPT does not impose general cytotoxicity

Given the therapeutic potential of combining NAC and 8-CPT in the treatment of ALL, it was important to examine whether NAC/8-CPT also would kill normal cells. ALL cells belong to the B-cell lineage, and one of the normal cell types closely related to ALL cells are therefore circulating B lymphocytes. Accordingly, we isolated normal B cells from the peripheral blood of healthy blood donors. The resting B cells were stimulated by the polyclonal activators CpG-ODN and anti-RP105 as previously described (36), and the activated B cells were treated with NAC in combination with 8-CPT. As expected, the viability of the B cells was improved by stimulating the cells (Fig. 7*C*). Importantly however, the viability of the B cells was unaffected by further treatment with NAC in combination with 8-CPT. We could therefore conclude that the combination of NAC and 8-CPT does not impose a general cytotoxicity.

### The combination of NAC and 8-CPT delays the progression of ALL *in vivo*

**Figure 8:**
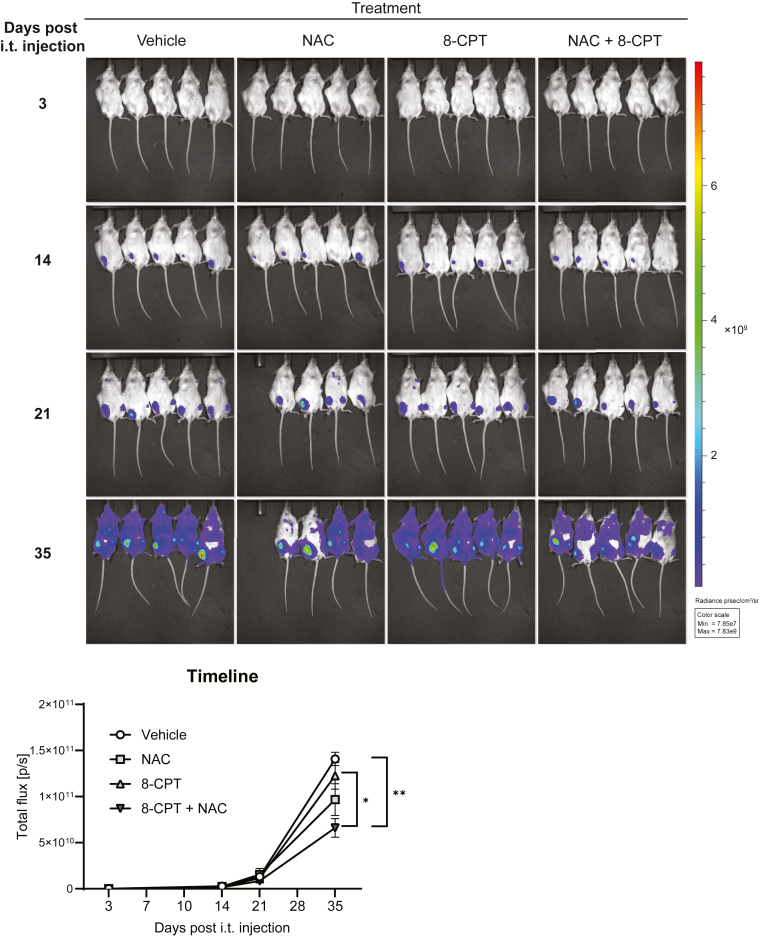
**8-CPT in the presence of NAC reduces the progression of ALL *in vivo*.** Xenograft REH mice were treated with NAC and 8-CPT as described in the Experimental procedures section. Development of leukemia was monitored by noninvasive *in vivo* imaging of luminescence from the mice. *Upper panel*: Luminescence image from xenograft REH mice taken at the indicated time points. *Lower panel*: Xenograft luciferase activity (photons per second, p/s) over time (n = 4–5 mice per treatment group, ∗*p* < 0.01, one-way ANOVA).

In light of the enhancing effect of NAC on 8-CPT-mediated killing of ALL cells *in vitro* and *ex vivo*, we here assessed the impact of NAC and 8-CPT on the progression of leukemia in our previously established xenograft model of ALL in NSG mice (14). REH cells were lentivirally transduced with a firefly luciferase-EGFP vector in order to track the leukemic cells by luminescence after intratibial injection of the cells. We included five mice per treatment group, and the progression of leukemia was followed by *in vivo* imaging as described in the Experimental procedures section. In the NAC-treated group, one mouse died after 20 days of treatment. The leukemia developed gradually over close to 5 weeks, before we terminated the experiment as the mice displayed symptoms beyond a predetermined humane endpoint. In line with the results from the *in vitro* and *ex vivo* experiments, NAC enhanced the effects of 8-CPT and significantly reduced the progression of leukemia compared to the vehicle-treated mice (Fig. 8). The experiment was repeated with essentially similar results, except that the experiment ended after 20 days, due to mice in the control (vehicle) group developing symptoms that required termination.

## Discussion

In the present study, we have shown that the ROS scavenger NAC is able to turn EPAC analogs into potent killers of ALL cells both *in vitro* and *ex vivo* and that the combination of NAC and 8-CPT significantly delays the progression of ALL *in vivo*. The rationale behind the study was initially to reveal the mechanisms behind our recent findings that cAMP signaling, *via* ROS-induced autophagy, reduces DNA damage-induced apoptosis in ALL cells (27, 28). We had previously shown that the adenylyl cyclase activator forskolin alone had only a minor effect on the viability of ALL cells, but that it protected the cells from IR-induced cell death (7, 11, 12, 13, 27, 28). Furthermore, we had demonstrated that the ROS scavenger NAC had negligible effect on the viability of cells treated with forskolin alone, but that NAC opposed the effects of forskolin on both IR-induced autophagy and apoptosis (28). When we in the present study explored the effects of NAC on the cAMP analogue 8-CPT, we surprisingly found that NAC not only relieved the protecting effects of 8-CPT on IR-mediated apoptosis but that NAC had a pronounced effect on 8-CPT-induced cell death alone. Hence, at optimal concentrations, NAC and 8-CPT induced apoptosis in more than 80% of the ALL-derived REH cells after 24 h.

To address the differential effects of NAC on forskolin-*versus* 8-CPT-mediated cell death, we turned to the cAMP signaling pathway. By demonstrating that NAC had the same potentiating effect on the EPAC-selective agonist 2′O-Me as on 8-CPT, we concluded that the ability of NAC to enhance the effects of 8-CPT on killing of ALL cells, was most likely mediated *via* EPACs. This notion was strengthened by the inability of NAC to enhance the cell death induced by the PKA-specific cAMP analogues 8-Br-cAMP and Sp-8-Br-cAMP. The involvement of EPAC1 was further confirmed by knockdown experiments, showing that that a 50% reduction in EPAC1 significantly reduced the NAC/8-CPT-induced cell death. It is well established that cAMP generated by the adenylyl cyclase activator forskolin can mediate its effects *via* both PKA and EPACs (37). The differential effects of NAC on forskolin *versus* 8-CPT is therefore not evident, and we reasoned that the differential effects could be explained by different kinetics and/or compartmentalization of the generated cAMP signals. When addressing the kinetics of the generated cAMP levels, we showed that both 8-CPT and forskolin rapidly increased the intracellular levels of cAMP (within 15 min). However, whereas the cAMP levels remained elevated in the 8-CPT-treated cells for at least 24 h, the forskolin-induced cAMP levels were nearly back to background levels already after 2 h. These results could imply that the differential effects of NAC on forskolin-*versus* 8-CPT-induced cell death might indeed reflect differences in the kinetics of the cAMP signaling. However, we could rule out this possibility, by showing that REH cells treated with suboptimal concentrations of forskolin in the presence of the phosphodiesterase inhibitor IBMX were not killed by NAC, despite sustained elevated levels of cAMP. We therefore believe that spatiotemporal differences in the cAMP levels is a more likely explanation for the differential effects of NAC on cell death induced by the forskolin *versus* 8-CPT. There are numerous reports on the spatial dynamics of PKA signaling (reviewed in (38)), and it was recently revealed that the effects of various adenylyl cyclase-stimulating receptors result in distinct cAMP nanodomains that mediates spatiotemporal specificity influencing the downstream signaling (39).

We should also consider the possibility that forskolin in the present cell system may exert its effect independent of its adenylyl cyclase modulating ability (40). Forskolin is known to block voltage-gated potassium channels (41) and inhibit GLUT1 glucose transporter (42). Potassium ions are the most predominant intracellular cations, and loss of potassium ions has been reported to favor activation of caspases that lead to apoptosis (43, 44). Indeed, the depletion of potassium ions or overexpression of potassium channels has been linked to the induction of apoptosis (45, 46, 47). Forskolin may therefore induce adenylyl cyclase-independent blocking of potassium channels, which in turn may interfere with pro-apoptotic process initiated by increased cAMP levels in the presence of NAC. It should however be emphasized, that the effects of forskolin on cell cycle- and DNA damage-mediated responses in REH cells in general can be mimicked by PKA and/or EPAC activators (48, 49, 50).

In our attempts to explain how NAC could turn 8-CPT and other EPAC activators into highly potent killers of ALL-derived cell lines and primary ALLs, we ruled out the possibility that NAC mediated its effects *via* its ability to inhibit ROS and autophagy. Hence, NAC inhibited forskolin- and 8-CPT-induced autophagy and ROS levels to the same extent, and the ROS scavenger vitamin C did not enhance the 8-CPT-induced cell death. We therefore turned our attention to the regulation of calcium homeostasis. Persistent moderately elevated levels of calcium over time may result in apoptotic cell death (26). EPAC is known to activate phospholipase C (PLC) that mediates the breakdown of PIP2 into IP_3_ and DAG (19), and EPACs can also generate IP_3_ directly (25). The resulting IP_3_ generation usually induces a rapid burst of intracellular calcium, leading to downstream signaling events. In our experiments, 8-CPT alone induced a small rise in calcium levels after 2 min, but the elevation was only transient. Accordingly, 8-CPT alone did not induce cell death in ALL cells. Interestingly however, NAC has also been reported to increase intracellular levels of calcium, trough activation of calcium channels like Ca^2+^ release-activated Ca^2+^ channels, TRPC, low voltage activated T-type calcium channels, transient receptor potential vanilloid (TRPV) channels (34), as well as L-type calcium channels (51). In fact, TRPV2 activation has been reported to induce apoptosis in an *in vitro* model of bladder cancer (52), and L-type calcium channel activity has been shown to trigger apoptosis through cytoplasmic calcium overload (53). We did not detect any rise in calcium levels of NAC alone at either early or late time points. Importantly however, NAC together with 8-CPT resulted in a rise in intracellular calcium that persisted for at least 20 h. We therefore suggest that NAC turns EPAC activators into killers of ALL cells *via* EPAC-mediated calcium release from intracellular stores combined with NAC-mediated calcium channel activation (TRPV2 or L-type calcium channels). This powerful combination culminates in long term calcium overload and killing of the ALL cells by induction of apoptosis, and it could potentially be a new strategy for treating pediatric ALL patients.

Today, more than 90% of patients with ALL survive but often with life-limiting morbidity due to the late effects of the harsh treatment. The patients would therefore greatly benefit from new therapeutic strategies or strategies that would increase the therapeutic index of current DNA-damaging agents. To test the potential of NAC in combination with 8-CPT as a novel therapeutic strategy for treating ALL, we took advantage of our xenograft model of ALL in NSG mice. When following the progression of leukemia in the mice by *in vivo* imaging, we found that NAC again enhanced the effect of 8-CPT and thereby delayed the progression of leukemia. We have previously identified the cAMP signaling pathway as a putative target for improving the therapeutic index of DNA-damaging therapy of ALL (7, 11, 12, 27, 28). This notion was based on the following observations: (i) ALL cells are exposed to cAMP-generating PGE_2_ in the bone marrow (9, 10), (ii) cAMP generated in the ALL cells reduces the effects of DNA-damaging agents on apoptosis (7, 11, 12, 28, 48), (iii) the cyclooxygenase inhibitor indomethacin delays the progression of ALL in a xenograft model by reducing the production of PGE2 (14), and (iv) the inhibitory effect of cAMP signaling on DNA damage-induced cell death is limited to the leukemic cells and not to normal B cell precursors (13). In light of these findings, we tested the impact of NAC in combination with 8-CPT on DNA damage-induced cell death, both induced by IR, and the more therapeutically relevant drug doxorubicin. It turned out that NAC not only prevented the protecting effect of 8-CPT on DNA damage-induced apoptosis, but that it strongly enhanced both the IR- and doxorubicin-mediated killing of the ALL cells. Importantly, we revealed that the ability of NAC in combination with 8-CPT to kill ALL cells was not due to general cytotoxicity. Thus, we demonstrated that the viability of the closely related normal peripheral blood B cells was unaffected by the same concentrations of NAC and 8-CPT that efficiently killed the primary ALL cells.

The results of the present study suggest that the combination of NAC and 8-CPT might be a powerful novel strategy for treating pediatric ALL. Moreover, this combination of drugs may also prove to limit the devastating side effects of DNA-damaging treatment of ALL, by its ability to enhance the efficacy, and thereby potentially increase the therapeutic index, of DNA-damaging agents.

## Experimental procedures

### Reagents and antibodies

Forskolin (#F6886), doxorubicin (#D1515), propidium iodide (PI) (#P4170), Ruthenium Red (RR) (#557450) and EDTA (#03690) were purchased from Sigma-Aldrich. CYTO-ID Autophagy detection kit ver. 2.0 (ENZ-KIT175) was purchased from Enzo Life Sciences. 8-CPT-cAMP (#C010-500) and 8-pCPT-2′-O-Me-cAMP (#C041-25) were obtained from BioLog, whereas N-acetyl-L-cysteine (#A9165), Fluo-3 (F1242), Indo-1 (I1223) and CellROX Green Oxidative Stress Reagent (C10444) were purchased from ThermoFisher. JC-1 (#420200) stain was purchased from Calbiochem. BAPTA-AM (#ab120503) and the EPAC1 rabbit monoclonal antibody (#ab109415) were obtained from Abcam, and the antibody was used at a final dilution of 1:1000. The GAPDH rabbit monoclonal antibody (#5174) and the rabbit polyclonal antibody recognizing cleaved-PARP (#9541S) were both purchased from Cell signaling technology, and they were used at a final dilution of 1:5000 and 1:1000, respectively.

### Cell culturing and primary cell isolation

The B cell precursor acute lymphoblastic leukemia cell lines REH (54), NALM-6 (55), and 697 (56) were maintained as previously described (27) at a density between 2 × 10^5^ and 1 × 10^6^ cells/ml in RPMI1640 supplemented with 10% heat inactivated fetal bovine serum, 125 U/ml penicillin, and 125 μg/ml streptomycin. Primary ALL cells were isolated from bone marrow aspirates of newly diagnosed ALL patients in line with previously established procedure (11). The expression of CD19 and CD10 on isolated primary ALL cells was determined by flow cytometry analyses by antibodies directed against CD19 (MACS Miltenyi Biotec #130-091-328) and CD10 (BioLegend #312218) as previously described (11). Collection of bone marrow aspirates from children diagnosed with ALL was performed after informed consent by parents, in accordance with the Declaration of Helsinki. Sample collection was approved by the Regional Ethics Committee of Norway region South-East C (REK 2014/883).

### DNA damage induced by IR or doxorubicin

DNA damage was induced by either exposing the cells to IR using a X-Strahl RS320 X-ray irradiator at a rate of 3.9 Gy/min, or by treating the cells with 100 nM of doxorubicin (Sigma-Aldrich). The two DNA-damaging agents inflicted similar levels of cell death, as measured by flow cytometry analyses of forward scatter (FSC) and side scatter (SSC). Due to the interference of doxorubicin with analyses of cell death by PI-staining and autophagy by CYTO-ID-staining (see below), we generally used IR as the preferred DNA-damaging agent.

### Western blot analysis

Cells were lysed in radioimmunoprecipitation (RIPA) buffer supplemented with aprotinin, as previously described (57). SDS polyacrylamide gel electrophoresis (SDS-PAGE) and western immunoblot analyses were performed as previously described (27).

### Flow cytometry analyses of cell death, autophagy, and ROS levels

All flow cytometry analyses (except Indo-1 analyses of calcium flux, see below) were performed on a NovoCyte (Acea Biosciences Inc) equipped with three lasers (488, 405, 640 nm) and 13 detection channels. For cell death analyses, cells were incubated with PI (20 μg/ml) for 10 min at 4 °C, and the incorporation of PI-staining of the cells was detected in the 572/28 channel. Changes in the mitochondrial membrane potential were determined by incubating the cells with JC-1 (15 μg/ml) at 37 °C for 15 min before analysis (excitation: 405 nm, detection 585/40 nm and 530/30 nm) (32). Staining of autophagosomes was performed by using the CYTO-ID Autophagy detection kit according to manufacturer’s protocol (excitation 488 nm, detection 530/30 nm). Levels of reactive oxygen species (ROS) were measured by using the CellROX Green Oxidative Stress Reagent according to the manufacturer’s protocol. Essentially, the cells were incubated with CellROX (5 μM) for 30 min at 37 °C followed by flow cytometry analysis (excitation 488 nm, emission 530/30 nm).

### Analyses of calcium flux and cAMP levels

For analyses of calcium flux by Fluo-3, the cells were incubated with Fluo-3 (10 μM) for 30 min in the dark at room temperature (RT). The cells were resuspended in HBSS buffer (Sigma, H6648-500 ml) supplemented with 1 mM glucose, followed by 30 min incubation at RT in the dark. Cells were analyzed by flow cytometry in the 530/30 channel. As an alternative method for analyses of calcium flux, the cells were incubated with Indo-1 (2.5 μM) for 30 min at 37 °C in the dark, followed by resuspention in fully supplemented RPMI1640 and flow analysis. As a positive control for calcium induction, we used the calcium ionophore ionomycin (58). The analyses were performed on BD LSR II flow cytometer equipped with a 20 mW 355 nm laser for excitation of Indo-1, and a 488 nm laser for light scattering. Indo-1 fluorescence was measured in two channels, 405/20 and 510/20. The ratio between these channels reflects intracellular free calcium concentration and is independent of how much Indo-1 is loaded in each individual cells. Intracellular levels of cAMP were measured by competitive ELISA (Abcam, ab23485), essentially following the instructions of the manufacturer by including the acetylation step.

### siRNA transfection

EPAC1 was knocked down by siRNA in the ALL cell line NALM-6. To this end, a total of 3 × 10^6^ NALM-6 cells were transfected with small-interfering RNA (siRNA) by using a nucleofector device (Amaxa Biosciences) and the Nucleofector Kit T (Lonza, #VVCA-1002) according to the manufacturer’s instructions and using the program C-005. For the knockdown of EPAC1, we used 53 nM of ON-TARGETplus Human RAPGEF3 siRNA SMARTPool (L-007676-00-0010), which is a cocktail of four different siRNAs with the target sequences: CGUGGGAACUCAUGAGAUG, GGACCGAGAUGCCCAAUUC, GAGCGUCUCUUUGUUGUCA, CGUGGUACAUUAUCUGGAA. Equimolar concentration of a non-targeting siRNA (D-001810-01-05) was used as control. All siRNAs were obtained from Dharmacon. After transfection, the cells were incubated for 72 h before further treatments were initiated.

### Lentiviral transduction of REH cells

REH cells were lentivirally transduced as previously described (14). In brief, cells (5 × 10^5^ cells/well) were seeded in 48-well plates in the culture medium. Lentiviral concentrates were added to the cells, and spinfection was performed by centrifuging the plates at 900*g* for 50 min at 34 °C. The plates were then incubated at 37 °C and 5% CO_2_ in a humidified atmosphere for 2 days before removing the viral particles by 2 repeated washes at 300*g* for 10 min at 4 °C. A small aliquot was taken from the cells to analyze the amount of EGFP^+^ cells by flow cytometry, and the remaining cells were subjected to intratibial (IT) injection into NSG mice.

### Mouse model of ALL

Murine xenograft models of ALL were established in 6 to 8 weeks old NOD-*scid* IL2R γ^null^ (NSG) mice (The Jackson Laboratory) by intratibially (i.t.) injecting lentivirally transduced ALL cells (2 × 10^5^ cells) expressing EGFP and fLuc (14, 28). The proximal end of the tibia was exposed as the knee was kept in a flexed position. A 23 G needle was used to drill a hole into the tibia before injecting the ALL cells (40 μl per animal) using a 31G insulin syringe. The procedure was performed under deep isoflurane anesthesia and the animals received local and general analgesics both during the procedure and a maintenance dose 6 to 8 h later. Osmotic pumps containing 8-CPT-cAMP (250 mM) or vehicle (methanol) were subcutaneously implanted into the mice 1 to 2 days after i.t. injection. Establishment of the xenograft model and cancer progression was determined by bioluminescence imaging using an IVIS Spectrum CT from PerkinElmer 1 to 2 times per week. NAC (1 mg/ml) was administered in the drinking water from the same day as the pump implantation and exchanged three times per week. Animals that had high leukemia burden or displayed symptoms of suffering were humanely euthanized. Animals were kept under appropriate housing conditions with food and water ad libitum. Experiments were approved by The Norwegian Food Safety Authority (FOTS id 26736).

### Statistical analyses

Statistical analyses were performed by using the GraphPad Prism8 software. To determine statistically significant differences between two groups, the paired *t* test was utilized, while comparisons between three or more groups ANOVA testing was performed. Statistical significance was set at *p*-value below 0.05. Data are presented as the mean (vertical graphs), with horizontal bars indicating the standard deviation (SD). Small circles indicate the individual data points. Each experiment was repeated at least three times as indicated in the figure legends.

## Data availability

All data relevant to this study are included within this manuscript.

## Conflict of interest

The authors declare that they have no conflicts of interest with the contents of this article.
